# Effect of acupuncture for diarrhea-predominant irritable bowel syndrome: study protocol for a randomized clinical trial

**DOI:** 10.1186/s13063-022-06639-5

**Published:** 2022-08-26

**Authors:** Ling-Yu Qi, Jing-Wen Yang, Shi-Yan Yan, Yan-Fen She, Hui Hu, Ying Li, Li-Li Chi, Bang-Qi Wu, Jian-Feng Tu, Li-Qiong Wang, Cun-Zhi Liu

**Affiliations:** 1 International Acupuncture and Moxibustion Innovation Institute, School of Acupuncture-Moxibustion and Tuina, Beijing University of Chinese Medicine, Beijing, 100029 China; 2 School of Acupuncture-Moxibustion and Tuina, Hebei University of Chinese Medicine, Shijiazhuang, 050299 China; 3 Department of Acupuncture and Moxibustion, Dongfang Hospital, Beijing University of Chinese Medicine, Beijing, 100029 China; 4 School of Graduate, Chengdu University of Traditional Chinese Medicine, 610075 Chengdu, China; 5 Department of Spleen and Stomach, Shandong University of Traditional Chinese Medicine Affiliated Hospital, Jinan, 250011 China; 6 National Acupuncture and Moxibustion Clinical Medical Research Center, the First Teaching Hospital of Tianjin University of Traditional Chinese Medicine, Tianjin, 300193 China

**Keywords:** Acupuncture, Diarrhea-predominant irritable bowel syndrome, Randomized controlled trials, Protocol

## Abstract

**Background:**

Diarrhea-predominant irritable bowel syndrome (IBS-D) is the most common subtype of IBS. Acupuncture is commonly used to treat IBS-D, but its effect is uncertain because of the poor quality of prior studies. This trial aims to evaluate the efficacy and safety of acupuncture treatment for IBS-D through comparisons with sham acupuncture.

**Methods/design:**

This is a large-scale, multi-center, randomized, two-arm interventional clinical trial. Participants will take part in a total of 20 weeks of study, which contained 3 phases: 2-week screening, 6-week treatment, and 12-week follow-up. Based on the composite response rate of the primary endpoint in our pilot study (a sham acupuncture response rate of 27% and a true acupuncture of approximately 45%), 280 randomly allocated participants were planned. Eligible participants will be randomly assigned to the true acupuncture group and sham acupuncture group according to a ratio of 1:1, and a total of 15 sessions of treatment overall 6-week treatment period will be brought. The primary endpoint is a composite response rate at week 6, and the responder is defined as who responses in both abdominal pain intensity and stool consistency. Furthermore, composite response rates at other weeks, IBS Symptom Severity Scale, IBS Quality of Life, Adequate Relief scale, and individual IBS symptoms (abdominal pain, bloating, stool frequency) are chosen as secondary endpoints.

**Discussion:**

This trial may provide high-quality evidence for the efficacy and safety of acupuncture in the treatment of IBS-D. The results of this study will be published in peer-reviewed journals.

**Trial registration:**

Chinese Clinical Trial Registry: ChiCTR2100044762. Registered on 26 March 2021.

## Background

Irritable bowel syndrome (IBS) is a globally prevalent disorder characterized by persistent or intermittent abdominal pain, bloating, changes in stool form and frequency, and defecation habits. Although IBS is non-fatal, the symptoms impair quality of life and social functioning, which lead to a healthcare burden. The annual direct and indirect costs associated with IBS are estimated to be up to billions or more in several countries [1–3]. In the absence of distinct biomarkers, IBS is typically diagnosed according to the patients’ self-reported symptoms, such as the Rome IV or Rome III criteria. According to a systematic review and meta-analysis on the global prevalence of IBS in 2020 [4], diarrhea-predominant IBS (IBS-D) is the most common subtype when the Rome IV criteria were applied. Routine clinical treatments for it tend to improve the most troublesome symptoms, such as abdominal pain and bowel habit. Among them, antidiarrheals and antispasmodics were used as first-line therapies for predominant stool pattern improvement in IBS, but most randomized controlled trials of these drugs are outdated and hampered by suboptimal methodology and heterogeneous patient selection, and the occurrence of side effects and adverse events greatly reduced the therapeutic effect and patients’ satisfaction, which resulted in a higher demand for alternative therapies [5].

Acupuncture is considered as a beneficial alternative treatment for functional gastrointestinal disorders [6]. Although whether acupuncture is an alternative treatment for IBS still remains controversial [7–11], previous studies have shown that acupuncture may affect IBS from the perspectives of the brain-gut axis, gastrointestinal motility, and visceral hypersensitivity [12, 13]. Furthermore, the recent clinical randomized trial supported that acupuncture may be more effective than pinaverium bromide for the treatment of IBS-D, with effects lasting up to 12 weeks [14]. Regrettably, the efficacy endpoints of most clinical trials of acupuncture for IBS often do not meet the current recommendations from the US Food and Drug Administration (FDA) or the European Medicines Agency. We have conducted a pilot clinical randomized trial with a minimum sample size (*n* = 90) in 2020 using efficacy endpoints that meet the current recommendations from the FDA [15]. For the primary endpoint, the composite response rates at week 4 between true acupuncture (specific acupoints and non-specific acupoints) and sham acupuncture (non-acupoints) were 46.67% and 26.67%, respectively, and the line charts trends of all endpoints of specific acupoints acupuncture are consistent and better than sham acupuncture. However, the effective response rates of different acupuncture groups and sham acupuncture group have no statistical significance, and we considered that the insufficient sample size cannot be ignored. Therefore, a large-sample, multicenter randomized controlled trial with high methodologic quality was considered necessary.

Accordingly, we designed the large-scale trial in order to provide substantive evidence on acupuncture as a reliable option. The primary objective is to evaluate the efficacy of acupuncture for the treatment of IBS, and the second objective is to assess the safety of the present acupuncture protocol.

## Methods/design

### Trial design

**Fig. 1:**
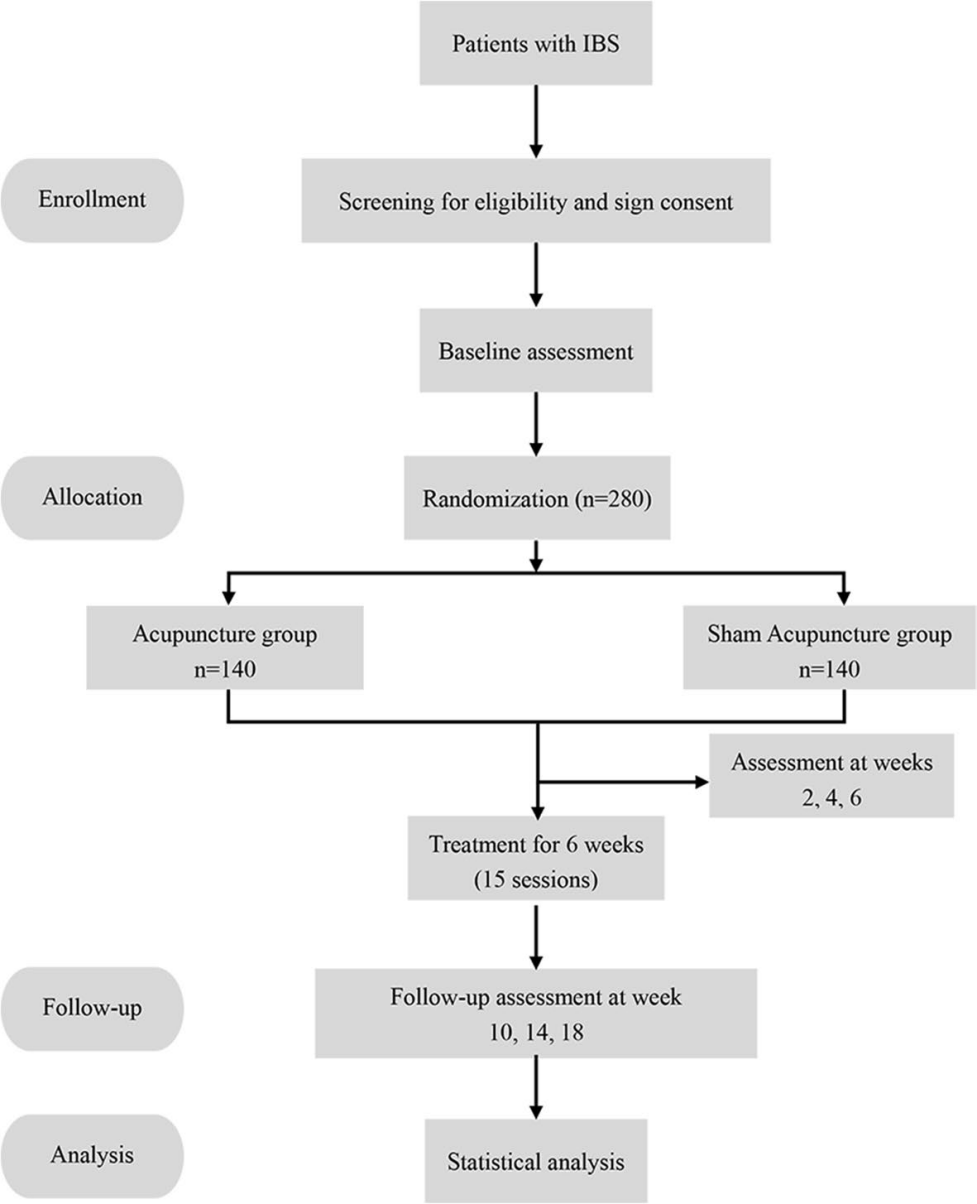
The flow diagram of this trial

This is a large-scale, multi-center, randomized, two-arm interventional clinical trial (Fig. 1). Participants will take part in the 20 weeks study which included 3 phases: 2-week screening (weeks − 2, − 1), 6-week treatment (weeks 1–6), and 12-week follow-up (weeks 7–18). Participants will be recruited in 6 grade III level A hospitals in China: (I) the Affiliated Hospital of Hebei University of Chinese Medicine, (II) the Teaching Hospital of Chengdu University of Traditional Chinese Medicine, (III) the Affiliated Hospital of Shandong University of Traditional Chinese Medicine, (IV) the First Teaching Hospital of Tianjin University of Traditional Chinese Medicine, (V) the Dongfang Hospital Beijing University of Chinese Medicine, and (VI) Shaanxi Provincial Hospital of Chinese Medicine. The study protocol was approved by the Medical Ethics Committee of Beijing University of Chinese before the study started. All personnel participating in this trial will be uniformly trained on the content of the trial.

### Participants

Participants will be recruited by the clinical recruiters using advertisements (e.g., flyers in physician offices, articles on WeChat official accounts). Data of the trial will be collected by the REDCap electronic data capture (EDC) system [16]. Every participant will sign an informed consent form before randomization. Patients will be considered to have dropped out if they did not complete 80% of the total treatment sessions.

#### Inclusion criteria

The following are the inclusion criteria:

(I) Aged between 18 and 75 years (either sex)
(II) Fulfilled Rome IV diagnostic criteria for IBS-D
(III) Type 6 or 7 of the Bristol Stool Form Scale appeared for at least 4 days and type 1 or 2 appeared for less than 4 days in the last 2 weeks; the average score of daily abdominal pain was ≥ 3 in the last week
(IV) No treatment of acupuncture in the last 6 months
(V) No use of antidepressant or IBS medication within 2 weeks before treatment, including traditional Chinese medicine (TCM) or proprietary Chinese medicine, antidiarrheal, antispasmodic, intestinal antibiotics, probiotics, and so on

#### Exclusion criteria

The following are the exclusion criteria:

(I) Participants with inflammatory bowel disease, microscopic colitis, celiac disease, Crohn’s disease, and other organic bowel diseases (age ≥ 50 years or have the following alarm signs that will be required to provide colonoscopy report within nearly 2 years: unexplained weight loss (weight loss > 10% within 3 months), hematochezia caused by non-hemorrhoids or anal fissure, nocturnal diarrhea, family history of colorectal cancer)
(II) Diabetes mellitus and abnormal thyroid function
(III) Severe acute or chronic organic diseases and kidney or liver diseases
(IV) History of previous abdominal surgery (appendectomy, hemorrhoidectomy, or polypectomy greater than 3 months post-surgery is allowed)
(V) Pregnancy or lactation
(VI) History of alcohol and drug abuse
(VII) Participate in other clinical trials

### Interventions

The true acupuncture (TA) group and sham acupuncture (SA) group will be set in this trial. The acupuncturists will be required to have a minimum licensed time of 3 years. Participants will receive 15 sessions of treatment overall 6-week treatment duration. Treatment will be given 3 times a week for the first 3 weeks (once every other day) and 2 times a week for the last 3 weeks (once every three days). Single-use sterile needles (0.30 mm in diameter and 40 mm in length or 0.30 mm in diameter and 25 mm in length; Hwato, Suzhou, China) will be used in the TA group. Blunt-tipped placebo needles will be used (similar to the Streitberger design) in the SA group which can provide participant-blinding effects with a similar appearance to conventional needles but no skin penetration [17, 18]. Adhesive pads will be placed on puncture points in both groups. The use of blunt-tipped placebo needles and adhesive pads is to help maximize the blinding of participants in the SA group, and adhesive pads also have the function of fixing blunt-tipped placebo needles. The research manual will be developed prior to the trial, and uniform training will be provided to all study staff (including acupuncturists), in areas such as recruitment, evaluation of outcomes, positioning of acupuncture acupoints, and acupuncture manipulation. During the treatment phase, each sub-center will have 1–2 acupuncturists implementing both groups of the intervention. We will require the acupuncturist to be responsible for the complete treatment of each patient rather than requiring the acupuncturist to specialize in a particular group of treatments, so that patients will not be able to differentiate between the treatment groups through the acupuncturist. In addition, patients assigned to the placebo group will be offered a free acupuncture treatment at the end of the follow-up period if they wish.

### TA group

Five fixed acupoints and one of three optional acupoints will be chosen for the treatment of participants in the TA group (Table 1). Based on the specific acupuncture acupoint combinations theory of TCM in *Lingshu* [19], the acupuncture prescription was developed by reviewing the frequency of acupuncture points in the relevant literature and combining it with the opinions of acupuncturists. The different types of TCM syndrome further guide the selection of optional acupuncture acupoints, such as Taichong (LR3) for the syndrome of liver depression and spleen deficiency, Sanyinjiao (SP6) for the syndrome of spleen deficiency and dampness obstruction, and Neiting (ST44) for the syndrome of spleen-stomach damp-heat. At the first visit, the acupuncturist will diagnose the patient’s TCM syndrome through questioning, tongue examination, and pulse and then select the appropriate optional acupoint. After sterilization, the acupuncturist will insert single-use sterile needles into the deep tissue layers through adhesive pads of acupoints. Following needle insertion, small, equal manipulations of twirling, lifting, and thrusting will be performed on all needles to reach *deqi* (a component sensation, including soreness, numbness, distension, and heaviness). The retention time of single-use sterile needles will be 30 min, during which the operation of *deqi* will also be performed every 10 min during the needle retention period.

**Table 1:** Locations of acupoints for the TA group

	Acupoint	Location
Fixed acupoints of the TA group	*Tianshu* (ST25)	On the horizontal line of the navel, 2 cun^a^ beside the anterior midline
	*Zhongwan* (RN12)	On the anterior midline of the upper abdomen, 4 cun superior to the navel
	*Guanyuan* (CV4)	On the anterior midline of the abdomen, 3 cun inferior to the navel
	*Zusanli* (ST36)	3 cun directly below ST35 and one fingerbreadth lateral to the anterior border of the tibia
	*Shangjuxu* (ST37)	On the anterolateral aspect of the leg, 6 cun inferior to the ST35, and one fingerbreadth lateral to the anterior border of the tibia
Optional acupoints of the TA group	*Taichong* (LR3)	In the depression anterior to the junction of the first and second metatarsal bones
	*Sanyinjiao* (SP6)	On the tibial aspect of the leg, posterior to the medial border of the tibia, and 3 cun superior to the prominence of the medial malleolus
	*Neiting* (ST44)	On the instep, between the second and third toes of the red and white flesh behind the webbed margin

^a^1 cun (≈ 20 mm) is defined as the width of the interphalangeal joint of the participant’s thumb

### SA group

The positioning of non-acupoints adheres to the following two principles: first, the sites of non-acupoints are selected away from the meridians or conventional acupoints; second, the distribution areas of non-acupoints are similar to that of the acupuncture acupoints to avoid patients differentiating groups by the location of acupoints. Five non-acupoints will be selected for the treatment of the SA group (Table 2). Unlike the TA group, the SA group will not perform manual operations for needles after needle insertion and pretend to perform the manual operation of *deqi* every 10 minutes during needle retention time.

**Table 2:** Locations of non-acupoints for the SA group

Non-acupoint	Location
Non-acupoint 1	On the abdomen, 2 cun^a^ superior to anterior superior iliac spine, between the gallbladder meridian and the spleen meridian
Non-acupoint 2	On the abdomen, 2 cun inferior to the navel, 1 cun beside the anterior midline, between the kidney meridian and the stomach meridian
Non-acupoint 3	On the lateral aspect of the leg, 3 cun inferior to GB34, between gallbladder meridian and bladder meridian
Non-acupoint 4	On the leg, 2 cun superior to the medial malleolus, in the middle of the medial tibia, between the liver meridian and the spleen meridian
Non-acupoint 5	On the leg, the midpoint of the line between GB40 and ST41, between the gallbladder meridian and the stomach meridian

^a^1 cun (≈ 20 mm) is defined as the width of the interphalangeal joint of the participant’s thumb

All participants will be advised to keep their routine diets and lifestyles during the study procedure. Loperamide (Imodium, Xian Janssen Pharmaceutical Ltd., China), will be used as a rescue medication under the guidance of gastroenterologists whenever necessary. The medication status and other non-irritable bowel syndrome drug applications of participants will be strictly recorded during the trial.

### Sample size

The sample size calculation was based on the composite response rate of the primary endpoint in our pilot study (not yet published). The results of our pilot study showed that a sham acupuncture response rate of 27% and a true acupuncture of approximately 45% were estimated for the primary endpoint. A sample size of 222 evaluable participants was calculated to be needed for 80% power. With an estimated dropout rate of 20% and the balance of recruitment quotas in 5 sites, 280 randomly allocated participants were planned.

### Randomization, sequence generation, and blinding

Eligible participants will be randomly assigned to the TA group and SA group according to the ratio of 1:1 by central stratified block randomization in the REDCap EDC system with a dynamic block size. The randomization sequence was created by a biostatistician who did not participate in this trial. Recruiters will be responsible for registering the participants, and acupuncturists will assign the participants to the intervention. The different REDCap EDC system permissions depend on the role of the personnel. Only the acupuncturist and his or her assistant can obtain the randomization grouping information. The clinical recruiters, outcome assessors, data managers, and statisticians will be blinded to the group allocation.

### Outcomes and follow-up

The primary endpoint is a composite response rate at week 6 in accordance with the FDA recommendations. A participant is defined as a composite responder when he or she is a responder in both abdominal pain intensity and stool consistency. The definition of abdominal pain intensity responder is defined as a decrease in the weekly average of worst abdominal pain in the past 24 h score of at least 30% compared with baseline. Stool consistency responder is defined as a decrease of at least 50% in the number of days per week with at least one stool that has a consistency of type 6 or 7 compared with baseline.

The secondary endpoints also include composite response rates at other weeks (weeks 2, 4, 10, 14, and 18). The IBS Symptom Severity Scale (IBS-SSS) is used to evaluate the overall symptoms of IBS, which comprises 5 domains (severity of abdominal pain, frequency of abdominal pain, severity of abdominal distension, degree of dissatisfaction with defecation habits, and interference with the quality of life) that are scored from 0 to 100, and total score range 0 to 500. A decrease of 50 points is considered as a minimal clinically important difference [20]. The IBS Quality of Life (IBS-QoL) comprises 34 IBS-specific items which divide into 8 variables: health worries, food avoidance, body image, dysphoria, interference with activity, social reactions, sexual activity, and relationships [21]. The IBS-QoL score will be transformed into a 0–100 scale using the following formula: (the actual raw score − the lowest possible score)/possible score range × 100. The Patient Health Questionnaire-9 (PHQ-9) is a diagnostic instrument for common mental disorders, of which scores range from 0 to 27 and the mental state is inversely proportional to the total score of it [22]. Individual IBS symptoms (abdominal pain, abdominal bloating, stool frequency) will also be collected and analyzed. The Adequate Relief (AR) scale will be used to confirm whether participants have an adequate relief by a two-category (yes or no) answer. IBS-SSS, IBS-QoL, PHQ-9, AR scale, and individual symptoms will be evaluated at weeks 2, 4, 6, 10, 14, and 18.

**Fig. 2:**
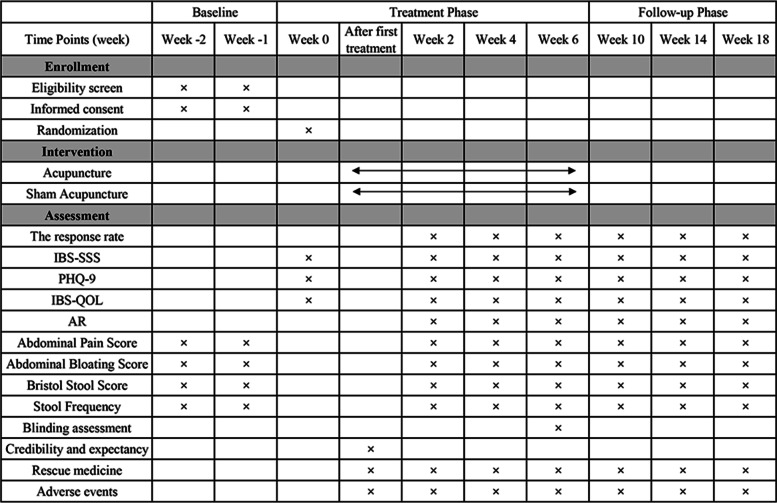
Schedule of enrollment, intervention, and assessments

For blinding assessment, all participants will be asked to guess which treatment they received at the end of the last treatment in week 6. Additionally, the Credibility/Expectancy Questionnaire within 5 min will be used to evaluate the credibility and expectancy of participants after the first treatment [23]. The schedule of enrollment, intervention, and assessments is shown in Fig. 2. The incidence of AEs and any use of loperamide will be recorded at 6-week treatment and 12-week follow-up.

### Human body data retention

Considering that the different types of testing instruments in different hospitals will affect the results of sample analysis, we will carry out the sample retention work from Dongfang Hospital Beijing University of Traditional Chinese Medicine. Stool samples and functional magnetic resonance imaging (fMRI) data at baseline and at the end of the intervention will be collected with the participants’ consent. It is well established that in IBS, communication between the gut and the brain is impaired, resulting in bowel movement disorders, visceral hypersensitivity, and altered central nervous system processing [24]. Through neuroimaging technology, the cooperative regulation mechanism of the “gut-brain” axis can be explored, which provides an important theoretical support for the study of the acupuncture effect. Based on the literatures, we will focus on the pain inhibition and conduction pathway and the emotion regulation pathway [25]. The MRI details were acquired via a 3.0-T Siemens scanner (Skyra, Siemens, Erlangen, Germany), and the data can be divided into two main categories including rest-state fMRI (rs-fMRI) and diffusion tensor imaging (DTI). Additionally, a gut microbiota analysis will be performed to assess the species abundance spectra or microbiota diversity. Bacterial DNA will be investigated by 16S rRNA gene sequencing targeting the hypervariable V3–V4 regions [26].

### Data management and monitoring

All researchers will be required to strictly protect the individual privacy of the participants. The electronic case report form (eCRF) will be used to input the general information and evaluation content related to the trial. Dynamic management will be performed to ensure complete, timely, and accurate data collection using the verification function in eCRF, and the researchers will no longer be able to modify the content of eCRF, because the database will be locked by the data management team when the trial is completed. In order to facilitate the elderly or other participants who are inconvenient to use electronic devices, we will also provide corresponding paper materials (e.g., defecation diary cards). Both paper files and electronic documents will be preserved for at least 5 years after publication. If readers and reviewers have any questions, they can contact the corresponding author for access to the original data.

Experts in acupuncture, gastroenterology, methodology, and statistics will review and revise the protocol. Before the trial, all researchers will be trained on standard operating procedures (such as screening patients, acupuncture, filling in eCRF, evaluation results, and data management). Online monitoring and on-site monitoring will be adopted in this trial. All modifications of the data can be traced through the eCRF. Appropriate communication will be maintained with the patients to strengthen their compliance and ensure data integrity. In addition, a Data Safety and Monitoring Board (DSMB) will be independently established to review and interpret the data of the trial. The board will review the progress of the trial, independently of the investigators, and decide whether the trial needs to be terminated early solely on the basis of adverse events.

### Statistical analyses

The following hypothesis will be tested simultaneously for the primary outcome:

- H0: There is no difference in the response rate between the TA and SA groups.
- H1: There is a significant difference in the response rate between the TA and SA groups.

All analyses related to the treatment efficacy will be based on the intention-to-treat (ITT) population, defined as all randomly assigned participants with baseline data. Safety analyses will be based on the safety population who have received at least one treatment after randomization and have safety indicator records. Missing data will be imputed using multiple imputation.

Continuous data will be represented by mean ± standard deviation (M±SD) or median combined with interquartile range, while categorical data will be represented by frequency, constituent ratio, and percentage. For the primary endpoint, the chi-square test will be used for the comparison of the two groups. For the secondary endpoints, Student’s *t* test or Wilcoxon rank-sum test will be used to assess the continuous variables between group comparisons, and the chi-square test or Fisher exact tests will be used to evaluate the categorical variables between the groups. The safety population will be used to analyze the incidence of AEs between the groups. The *P* value less than 0.05 will be considered as statistically significant. All analyses will be performed using SAS 9.3.

### Adverse event

Adverse events (AEs) will be appropriately assessed, managed, and recorded by the acupuncturists and related clinical specialists. We have set a specific questionnaire for recording AEs in real time by the REDCap EDC system. All AEs will be dealt with symptomatically. If the acupuncturist is unable to handle it, it is necessary to consult specialists of the corresponding discipline for consultation and treatment. Common acupuncture-related AEs include subcutaneous hematoma, continuous post-needling pain, and dizziness.

## Discussion

Although IBS has been extensively studied, its uncertain pathophysiology and unsatisfactory treatments prompted a growing demand for alternative treatments, including acupuncture. This large-scale trial will evaluate the efficacy and safety of acupuncture for the treatment of IBS.

For this trial, the application of blunt-tipped placebo needles, adhesive pads, and REDCap EDC system will enable the trial progress to better achieve the methodological demand for randomization, allocation concealment, and blinding of participants, clinical recruiters, outcome assessors, data managers, and statisticians. Meanwhile, we will assess the participant expectation and blinding which seems have an influence on acupuncture effects. Additionally, the primary endpoint which complies with the FDA standards will make the trial results more convincing. To the best of our knowledge, no prior RCT using FDA-recommended indicators to compare the effect between true acupuncture and sham acupuncture on IBS-D.

The intestinal microbiota is currently a popular area of research. Studies on intestinal microbiota of IBS found that patients with IBS had a higher abundance of the phylum Firmicutes and lower abundance of Bacteroides compared with healthy controls [27, 28], and the fecal microecological diversity of various subtypes of IBS patients was different [29]. In addition, Quigley et al. have suggested that the over-reproduction of intestinal microbiota may be an important factor in the induction of IBS [30]. Changes in the intestinal microbiota are closely related to IBS; however, there are no studies to confirm the regulatory effect of acupuncture on the intestinal flora of IBS. Moreover, symptoms of IBS are considered as disorders of the nervous system of the brain, and fMRI is an effective method to explore abnormal brain functions. Some researchers have used fMRI to investigate the potential mechanism of IBS, but the results are not completely consistent [12, 31]. Therefore, we will retain their stool samples and fMRI data according to the willingness of the participants to explore the possible mechanisms of acupuncture treatment of IBS.

There are several limitations in this trial. First, a previous study put forward that 6 sessions of treatment of acupuncture over 3 weeks may have been insufficient [32], so we chose the 15 sessions of treatment overall 6-week treatment period to achieve maximum effect from acupuncture. This may be more in line with actual clinical practice in China, but it may be difficult to implement in other countries due to treatment costs, commuting, and travel time. Therefore, we have made modifications for per week sessions (i.e., 3 or 2 sessions of treatment per week) to explore a standardized acupuncture protocol for IBS. Second, this trial failed to blind acupuncturists which may affect the effect of interventions between the groups. Third, although interventions in the SA group have no manual operation of skin penetration, they cannot eliminate the placebo effect completely, which should be taken into consideration when drawing conclusions.

### Trial status

This trial is currently recruiting patients.

### Registration

Chinese Clinical Trial Registry: ChiCTR2100044762. Registered on 26 March 2021.

### Protocol

There was no public or patient involvement in the design of the protocol. This is the second protocol version revised on 11 April 2022. The trial registrar will make changes to the content of the trial protocol (e.g., changes in eligibility criteria, analysis plans) in real time in the Chinese Clinical Trial Registry (http://www.chictr.org.cn/).

## Data Availability

Following the publication of this study, the authors will communicate the trial results to participants, healthcare professionals, the public, and other relevant groups. The corresponding author can be contacted to obtain data according to reasonable requirements.

## Abbreviations

REDCap: Research Electronic Data Capture
IBS-D: Diarrhea-predominant irritable bowel syndrome
IBS: Irritable bowel syndrome
FDA: Food and Drug Administration
EDC: Electronic data capture
TCM: Traditional Chinese medicine
TA: True acupuncture
SA: Sham acupuncture
IBS-SSS: IBS Symptom Severity Scale
IBS-QoL: IBS Quality of Life
PHQ-9: Patient Health Questionnaire-9
AR: Adequate Relief
fMRI: Functional magnetic resonance imaging
eCRF: Electronic case report form
DSMB: Data Safety and Monitoring Board
ITT: Intention-to-treat
M±SD: Mean ± standard deviation
AEs: Adverse events
